# Progressive Supranuclear Palsy-Primary Lateral Sclerosis Overlap Syndrome in a Patient with a *CYLD* Variant

**DOI:** 10.2478/bjmg-2026-0005

**Published:** 2026-10-14

**Authors:** NK Çiftçi, G Gürsoy, M Eser, HC Manazoğlu, NG Şirin, MH Yarar, N Pazarcı, HA Hanağası

**Affiliations:** Ümraniye Research and Training Hospital, Department of Neurology, Istanbul/Türkiye; Ümraniye Research and Training Hospital, Department of Medical Genetic, Istanbul/Türkiye; Istanbul University, Istanbul Faculty of Medicine, Department of Neurology, Clinical Neurophysiology Unit, Istanbul/Türkiye; Istanbul University, Istanbul Faculty of Medicine, Department of Neurology, Behavioral Neurology and Movement Disorders Unit, Istanbul/Türkiye

**Keywords:** *CYLD* gene, motor neuron disease, primary lateral sclerosis, progressive supranuclear palsy

## Abstract

**Objective:**

Progressive supranuclear palsy (PSP) is an atypical parkinsonian syndrome characterized by progressive axial rigidity, vertical gaze palsy, dysarthria, and dysphagia. This report describes a case of PSP-primary lateral sclerosis overlap syndrome associated with a *CYLD* gene variant.

**Case:**

We describe the case of a 59-year-old male patient who presented with slowing of movements and hypophonic speech. Neurological examination revealed hypomimia, dysarthric and hypophonic speech, restricted upward gaze, slow saccadic eye movements, rigidity and bradykinesia in all extremities, axial rigidity, brisk deep tendon reflexes, bilateral positive Hoffman’s sign, and bilateral extensor plantar responses. Brain MRI showed the “hummingbird sign” accompanied by bilateral cortico-spinal tract hyperintensity. MEP studies showed impaired excitability and conduction in the corticomotor system. Molecular genetic analysis identified a heterozygous G107A (c.320G>A; p.Gly107Asp) missense variant in the *CYLD* gene.

**Conclusion:**

Based on these findings, a diagnosis of PSP-Primary Lateral Sclerosis overlap syndrome was made. The contribution of the identified *CYLD* variant to this complex phenotype and its role in the neurodegenerative disease spectrum warrant further investigation.

## INTRODUCTION

Progressive supranuclear palsy (PSP) is an atypical parkinsonian syndrome characterized by progressive axial rigidity, vertical gaze palsy, dysarthria, and dysphagia [1]. The neuropathological hallmark of PSP is the abnormal accumulation of tau protein, predominantly affecting the pallidum, as well as the subthalamic nucleus, red nucleus, substantia nigra, pontine tegmentum, striatum, oculomotor nucleus, medulla, and dentate nucleus [2]. A disproportionate atrophy of the dorsal mesencephalon compared to other brainstem structures is a characteristic, yet insufficiently sensitive, diagnostic feature observed on magnetic resonance imaging (MRI) [3]. While the classic clinical presentation is known as Richardson’s syndrome, several subtypes associated with distinct neurodegenerative syndromes and findings have been described [4]. Herein, we report a unique case of PSP presenting with concomitant primary lateral sclerosis (PLS). Through this case, we review the relevant literature and discuss the clinicopathological overlap between these neurodegenerative entities, focusing on the potential shared or distinct genetic underpinnings suggested by the identification of a heterozygous G107A (c.320G>A; p.Gly107Asp) missense variant in the *CYLD* gene.

## CASE REPORT

A 59-year-old right-hand dominant male patient presented to our outpatient clinic with complaints of slowing of movements and hypophonic speech. The patient reported that four years before presentation, he had noticed difficulty articulating certain syllables. Within the following year, his speech progressively became more hypophonic and slurred. Over the last year, additional symptoms emerged, including generalized slowness of movement, a sensation of shuffling while walking, difficulty walking on uneven surfaces, episodes of falling both forwards and backwards, and loss of fine motor skills in his right hand. The patient described experiencing intense psychological stress around the onset of his symptoms and, believing his condition might be depression-related, he declined pharmacological treatment despite having been diagnosed with Parkinson’s disease by two separate neurologists.

His medical history was significant for depression, for which he was using escitalopram 10 mg tablet 1x1. He had no alcohol or substance abuse. His family history was notable for Alzheimer’s disease in his father (diagnosed at age 78, deceased at age 85).

On neurological examination, the patient was alert, cooperative, and fully oriented. He exhibited hypomimia. His speech was dysarthric and hypophonic, while his comprehension was intact. Direct and indirect light reflexes were present bilaterally. Eye movements were restricted on upward gaze, and saccadic eye movements were slow in all directions. The remaining cranial nerves were intact. Muscle strength was full in all four extremities. However, cogwheel rigidity was noted in the upper extremities, more pronounced at both wrists. Rigidity and bradykinesia were present in all limbs, accompanied by axial rigidity. The glabellar reflex showed normal habituation. Deep tendon reflexes were brisk in all four extremities. The patient exhibited a bilateral positive Hoffman’s sign and bilateral extensor plantar responses. His gait was characterized by small steps. Postural instability was present. During the examination, he displayed inappropriate episodes of crying and laughter, suggestive of pseudobulbar affect.

Routine complete blood count and biochemistry panels revealed no pathological findings. Brain magnetic resonance imaging (MRI) demonstrated the “hummingbird sign” accompanied by bilateral corticospinal tract hyperintensity (Figure 1). Cervical spine MRI showed straightening of the cervical lordosis and disc protrusion at a C6–7 level without nerve root compression. Contrast-enhanced computed tomography (CT) of the thorax and abdomen, performed for malignancy screening, showed no pathological findings. All tumour markers tested were within normal limits. The Montreal Cognitive Assessment (MoCA) test score was 27/30; he was unable to copy the cube, and his memory and delayed recall scores were 4/5.

**Figure 1.**
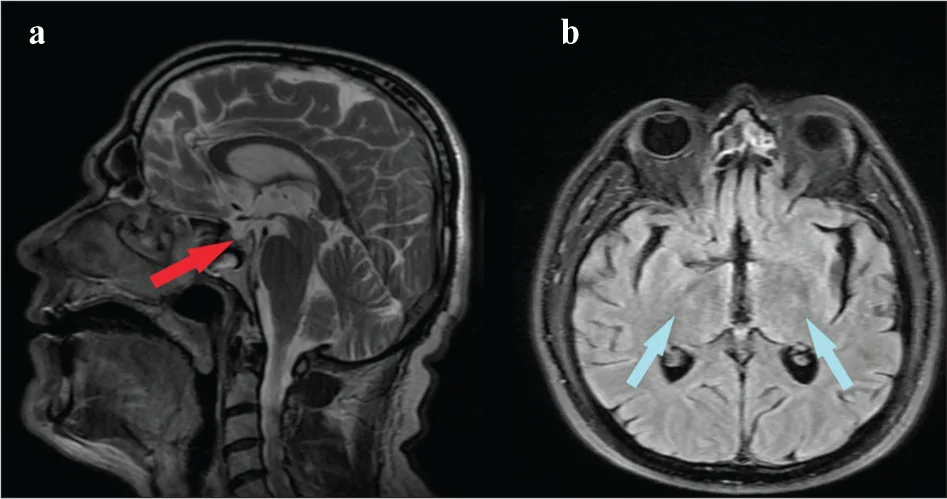
**a** - Midsagittal T2-weighted image demonstrating a hummingbird sign **b** - Axial FLAIR image demonstrating hyperintense corticospinal tract at the posterior limb of the internal capsule.

Electro neurophysiological studies revealed chronic neurogenic involvement in muscles innervated by the right L5 and left L4-S1 nerve roots. Motor evoked potential (MEP) studies, performed to evaluate upper motor neuron involvement, showed low excitability of the primary motor cortex corresponding to the left C8-T1 segments, which was in excitable. For the right C8-T1 segments, the corticomotor system exhibited impaired excitability and conduction. Studies performed for the bilateral L4–5 segments were normal.

Molecular genetic analysis for amyotrophic lateral sclerosis (ALS) revealed a heterozygous G107A (c.320G>A; p.Gly107Asp) missense variant in the *CYLD* gene.

In this patient, clinical, radiological, and electrophysiological findings were consistent with Progressive Supranuclear Palsy-Primary Lateral Sclerosis (PSP-PLS) overlap syndrome. Progressive axial rigidity, supranuclear vertical gaze restriction with slow saccades, postural instability with early falls, and the presence of a “hummingbird sign” on brain MRI supported PSP; while bilateral brisk deep tendon reflexes, bilateral Hoffman sign, bilateral extensor plantar responses, absence of marked muscle atrophy or fasciculations, impaired corticomotor excitability and conduction on MEP studies, and bilateral corticospinal tract hyperintensity on MRI supported PLS or a PLS-like upper motor neuron (UMN)-dominant syndrome. The presence of pseudobulbar affect further supported the UMN-dominant process.

Alternative diagnoses were carefully evaluated. Asymmetric cortical signs such as apraxia, cortical sensory loss, or alien limb phenomenon, cerebellar ataxia, autonomic dysfunction, and stridor, which are supportive of other Parkinson-plus syndromes were not detected. Lower motor neuron signs, which are necessary for amyotrophic lateral sclerosis (ALS), were also absent. Therefore, the PSP-PLS overlap phenotype remains the most consistent diagnosis.

Levodopa therapy was initiated. However, at the 6-month follow-up, it was discontinued due to lack of significant clinical benefit despite dose escalation (up to 600 mg/day).

## DISCUSSION

Progressive supranuclear palsy is a 4-repeat tauopathy. Historically characterized by vertical gaze palsy and early falls, clinicopathological studies have since revealed a spectrum of distinct phenotypic presentations [5]. The 2017 clinical criteria published by the International Parkinson and Movement Disorder Society (MDS) define four core clinical features—oculomotor dysfunction, postural instability, akinesia, and cognitive dysfunction—to categorize different PSP syndromes, thereby increasing diagnostic sensitivity [6]. Reported PSP subtypes in the literature include Richardson’s syndrome, PSP-parkinsonism, PSP-pure akinesia with gait freezing, PSP-progressive non-fluent aphasia, PSP-cerebellar ataxia, PSP-corticobasal syndrome, PSP-behavioural variant of frontotemporal dementia, PSP-primary lateral sclerosis, and PSP-pallidonigrolusian degeneration and axonal atrophy [7].

A study examining autopsy materials from 12 patients with concomitant PSP and PLS reported that 9 cases exhibited asymmetric limb involvement, similar to our patient. All cases demonstrated gliosis and neuronal loss in the motor cortex, along with cortical atrophy of the precentral gyrus and corticospinal tracts. Furthermore, degeneration was observed along the corticospinal pathway, extending from the motor cortex to the internal capsule and down to the level of the medullary pyramids. The authors noted that while all cases were defined as 4-repeat tauopathies, they did not fit neatly into any currently defined pathological or genetic diagnostic category. Given the significant overlap in pathological features with PSP, the cases were classified as atypical PSP. Levodopa/carbidopa combination therapy was attempted in these patients but yielded no clinical response, consistent with the lack of response observed in our patient [8]. In addition to this case series, the clinical presentations of two separate cases (featuring positive Babinski and Hoffman signs, asymmetric spasticity, etc.) were initially considered PLS, yet autopsy revealed 4-repeat tauopathy. These findings suggest that PSP can manifest even in the absence of gaze palsy, parkinsonism, or cognitive decline [9,10].

Both primary lateral sclerosis and PSP are neurodegenerative diseases that can present with motor and bulbar symptoms. They may co-occur, and differentiation can be challenging in the early stages. To aid differential diagnosis, a study utilizing diffusion tensor imaging (DTI) compared scans from 10 PSP patients with 3 patients initially diagnosed with PSP but later followed as PLS. It was found that the corticospinal tract was more affected in PLS patients, whereas the superior cerebellar peduncle was more affected in PSP patients. Based on this, a corticospinal tract/superior cerebellar peduncle ratio was calculated (p<0.2), proposed as a potential biomarker for differentiation [11]. In our patient’s cranial imaging, mesencephalic atrophy was observed alongside bilateral corticospinal tract hyperintensity. Neuroradiological and electrophysiological findings in our patient provide valuable supporting evidence for the proposed overlap phenotype; however, hummingbird sign and corticospinal pathway hyperintensity are not pathognomonic for PSP-PLS overlap and can be observed in other neurodegenerative conditions. Similarly, MEP abnormalities indicate upper motor neuron dysfunction but do not definitively differentiate PLS from other UMN syndromes.

Similar to PSP-PLS overlap cases, parkinsonism co-occurring with motor neuron diseases has also been reported. In an Italian cohort of 1,042 patients with motor neuron disease, 18 (1.7%) exhibited parkinsonian features. These patients had a later age of onset, more frequent accompanying oculomotor dysfunction and cognitive impairment, and a higher family history of dementia and parkinsonism. Among motor neuron disease subtypes, PLS showed the highest association with parkinsonism. Of these 18 patients, seven had concurrent Parkinson’s disease, while six were diagnosed with PSP. Unlike our patient, all cases with PLS-PSP overlap reported frontal cognitive impairment [12].

The heterozygous G107A (c.320G>A; p.Gly107Asp) missense variant in the *CYLD* gene identified in our patient has been associated with frontotemporal dementia (FTD) and/or amyotrophic lateral sclerosis (ALS) but is classified as a variant of uncertain significance. Literature reports describe the CYLD M719V variant in two independent FTD-ALS families, suggesting this genomic region may harbour a major locus for FTD-ALS [13]. While mutations that increase *CYLD* gene activity are rare, studies have shown they promote cytoplasmic localization of TDP-43, shorten axons, and disrupt the fusion of autophagosomes with lysosomes—a critical step in autophagy. Given these effects, *CYLD* is thought to play a central role in FTD-ALS pathogenesis [14]. In our patient, we identified a missense variant in a different region of the same gene. Given the rarity of the PSP-PLS overlap syndrome, no other genetic finding similar to our case has been reported in the literature. Consequently, the precise contribution of the identified gene to disease pathogenesis cannot be determined, and we can only speculate that it may be relevant to the spectrum of neurodegenerative disorders.

In conclusion, as illustrated by our case, clinicians should consider the possibility of upper motor neuron signs accompanying atypical parkinsonism, even if infrequent. The identified *CYLD* variant represents a potentially relevant finding in this unusual overlap phenotype. However, its pathogenic contribution remains uncertain and requires confirmation in additional cases and functional studies.
